# Identification of Piecemeal Degranulation and Vesicular Transport of MBP-1 in Liver-Infiltrating Mouse Eosinophils During Acute Experimental *Schistosoma mansoni* Infection

**DOI:** 10.3389/fimmu.2018.03019

**Published:** 2018-12-20

**Authors:** Felipe F. Dias, Kátia B. Amaral, Kássia K. Malta, Thiago P. Silva, Gabriel S. C. Rodrigues, Florence M. Rosa, Gisele O. L. Rodrigues, Vivian V. Costa, Hélio Chiarini-Garcia, Peter F. Weller, Rossana C. N. Melo

**Affiliations:** ^1^Laboratory of Cellular Biology, Department of Biology, Federal University of Juiz de Fora, Juiz de Fora, Brazil; ^2^Laboratory of Parasitology, Department of Parasitology, Microbiology and Immunology, Federal University of Juiz de Fora, Juiz de Fora, Brazil; ^3^Laboratory of Immunopharmacology, Department of Biochemistry and Immunology, Federal University of Minas Gerais, Belo Horizonte, Brazil; ^4^Center for Drug Research and Development of Pharmaceuticals, Federal University of Minas Gerais, Belo Horizonte, Brazil; ^5^Research Group in Arboviral Diseases, Department of Morphology, Federal University of Minas Gerais, Belo Horizonte, Brazil; ^6^Laboratory of Reproduction and Structural Biology, Department of Morphology, Federal University of Minas Gerais, Belo Horizonte, Brazil; ^7^Division of Allergy and Inflammation, Department of Medicine, Beth Israel Deaconess Medical Center, Harvard Medical School Boston, Boston, MA, United States

**Keywords:** schistosomiasis, eosinophil degranulation, major basic protein-1, granuloma, inflammation, liver, immunonanogold electron microscopy, piecemeal degranulation

## Abstract

Eosinophils have been long associated with helminthic infections, although their functions in these diseases remain unclear. During schistosomiasis caused by the trematode *Schistosoma mansoni*, eosinophils are specifically recruited and migrate to sites of granulomatous responses where they degranulate. However, little is known about the mechanisms of eosinophil secretion during this disease. Here, we investigated the degranulation patterns, including the cellular mechanisms of major basic protein-1 (MBP-1) release, from inflammatory eosinophils in a mouse model of *S. mansoni* infection (acute phase). Fragments of the liver, a major target organ of this disease, were processed for histologic analyses (whole slide imaging), conventional transmission electron microscopy (TEM), and immunonanogold EM using a pre-embedding approach for precise localization of major basic protein 1 (MBP-1), a typical cationic protein stored pre-synthesized in eosinophil secretory (specific) granules. A well-characterized granulomatous inflammatory response with a high number of infiltrating eosinophils surrounding *S. mansoni* eggs was observed in the livers of infected mice. Moreover, significant elevations in the levels of plasma Th2 cytokines (IL-4, IL-13, and IL-10) and serum enzymes (alanine aminotransferase and aspartate aminotransferase) reflecting altered liver function were detected in response to the infection. TEM quantitative analyses revealed that while 19.1% of eosinophils were intact, most of them showed distinct degranulation processes: cytolysis (13.0%), classical and/or compound exocytosis identified by granule fusions (1.5%), and mainly piecemeal degranulation (PMD) (66.4%), which is mediated by vesicular trafficking. Immunonanogold EM showed a consistent labeling for MBP-1 associated with secretory granules. Most MBP-1-positive granules had PMD features (79.0 ± 4.8%). MBP-1 was also present extracellularly and on vesicles distributed in the cytoplasm and attached to/surrounding the surface of emptying granules. Our data demonstrated that liver-infiltrating mouse eosinophils are able to degranulate through different secretory processes during acute experimental *S. mansoni* infections with PMD being the predominant mechanism of eosinophil secretion. This means that a selective secretion of MBP-1 is occurring. Moreover, our study demonstrates, for the first time, a vesicular trafficking of MBP-1 within mouse eosinophils elicited by a helminth infection. Vesicle-mediated secretion of MBP-1 may be relevant for the rapid release of small concentrations of MBP-1 under cell activation.

## Introduction

Eosinophils are innate immune cells with a broad distribution in tissues and notably associated with allergic and helminth parasitic diseases [reviewed in (1–3)]. Increase in the numbers of eosinophils has long been reported during the acute phase of schistosomiasis, a neglected tropical disease of great clinical and socioeconomic relevance (4–7). The etiological agents of human schistosomiasis are trematode worms of the genus *Schistosoma* with most species, including *Schistosoma mansoni*, the only one that occurs in the Americas, affecting mainly the liver and the intestines (8). Human infection with this parasite causes significant chronic morbidity with the development of a granulomatous reaction and severe tissue inflammation, which can lead to life-threatening hepatosplenomegaly [reviewed in (9)].

There is considerable debate on the role of eosinophils during schistosomiasis. It remains uncertain if eosinophils act as major effector cells against the parasite; as immunomodulators of the immune response; as participants in tissue homeostasis and metabolism, which could favor establishment and maintenance of parasitic worms in their hosts, or merely as operators in remodeling and clearance of debris following injury (7, 10–14).

Eosinophil responses to inflammatory and/or immunoregulatory situations are underlined by the ability of these cells to release granule-stored products, that is, to degranulate (15). Direct attention to events of eosinophil degranulation during schistosomiasis mansoni has been paid in earlier studies. Several authors showed *in vitro* that eosinophils, operating via antibody-dependent cytotoxicity, exert damage to schistosomula of S*. mansoni* (16, 17). This effect was attributable, at least in part, to degranulation and release of granule contents, especially to major basic protein (MBP), a typical cationic protein stored pre-synthesized in secretory (specific) granules, onto the surface of the parasite (18). In fact, the toxicity of MBP and Eosinophil Peroxidase/Eosinophil Protein X (EPO/EPX) has given support to the effector function of eosinophils in defense of the host to helminthes, although evidence for such importance have arisen just from *in vitro* data (reviewed in 13). Within *S. mansoni* egg-induced granulomas, there are well-defined clusters of eosinophils and other inflammatory cells embedded in a collagen-rich extracellular matrix around mature parasite eggs (7, 19), but there is a lack of clarity regarding the degranulation patterns of eosinophils and its significance in both humans and mouse models.

Here, we performed a comprehensive *in vivo* study to investigate the secretory processes involved in eosinophil degranulation during the acute phase of schistosomiasis mansoni in mice. By using conventional transmission electron microscopy (TEM), which is the only technique with resolution suitable to clearly identify and distinguish between different modes of cell secretion (20) and immunogold EM for detecting MBP-1 subcellular localization, we identified, for the first time, a major vesicle-mediated secretory process for MBP-1 release underlying the responses of eosinophils to the infection.

## Materials and Methods

### Experimental Infection in Mice

Swiss Webster mice aged 70 days were inoculated or not (12 mice per group) with a single inoculum of cercariae of *S. mansoni* (100 cercariae/mouse), LE strain. Cercariae were harvested from infected *Biomphalaria glabrata* snails, washed, counted, and injected subcutaneously into each mouse by an experienced technician. *S. mansoni* LE strain used in the experiments was originally isolated from a patient in Belo Horizonte, Brazil, and has been maintained in successive passages through *Biomphalaria glabrata* snails and hamsters (*Mesocricetus auratus*) at the Laboratory of Schistosomiasis (Department of Parasitology, UFMG, Brazil). Infected animals and respective uninfected controls from the same age were euthanized at 55 days of infection (acute phase) (5). Infection was confirmed by findings of parasite eggs in the rodent feces at week five of infection (21).

### Ethics Statement

This study was carried out in full accordance with all international and Brazilian accepted ethic guidelines and was approved by the Oswaldo Cruz Foundation Ethics Committee on Animal Use [CEUA-*Comissão de Ética no Uso de Animais*, under the protocol 32/2012). CEUA follows the Brazilian national guidelines recommended by the National Council for Animal Experimentation-CONCEA (*Conselho Nacional de Controle em Experimentação Animal*). Mice experimentally infected and uninfected controls were monitored daily for survival and well-being status (home cage evaluation, body condition, skin lesions, mobility and other general conditions) (22). No animals died prior to the experimental endpoint (55 days of infection).

### Collection of Samples

Experimentally infected animals and their respective uninfected controls were anesthetized, euthanized, and blood samples and organ fragments were collected for different studies as below. Animals were euthanized by exsanguination (full bleed) under deep anesthesia by cardiac puncture. The anesthetic protocols included ketamine (100 mg /mL) combined with acepromazine (10 mg /mL) at a ratio of 9:1 (dose of 0.15 mL/100 g body weight) (23). Blood samples were collected by cardiac puncture without anti-coagulant for enzyme determinations or with heparin for cytokine evaluations. Another group of infected and non-infected animals (*n* = 4 for each group) was euthanized in a CO_2_ chamber at the same day for peritoneal lavage (PL).

### Antibody Reagents

Rat monoclonal anti-mouse IgG2a MBP-1 (clone 14.7.4) (Lee Laboratories; Mayo Clinic, Scottsdale, AZ) whose MBP-1 specificity has been well validated in previous studies (24–26) and irrelevant isotype control monoclonal antibodies IgG (Abcam; Cambridge, MA) were used for the ultrastructural immunodetection studies at concentration of 20 μg/mL. The secondary antibody used for immunoEM was an affinity-purified goat anti-rat Fab fragment conjugated to 1.4-nm gold particles (1:100, Nanogold, Nanoprobes, Stony Brook, NY).

### Liver Enzymes Determinations

To evaluate the serum enzymes reflecting liver function, blood samples were centrifuged at 3000 rpm for 10 min and analyzed in a Roche Cobas Mira Plus Chemistry Analyzer (Roche Diagnostics®, IN, USA) as before (27). Assay Kits for measurement of the levels of aspartate aminotransferase (AST) and alanine aminotransferase (ALT) (Bioclin®, Belo Horizonte, MG, Brazil) were used according to the manufacturer's instructions. A total of 24 samples were evaluated from mice (12 from infected animals and 12 from uninfected of the same age). Results were expressed as units/liter (U/L).

### Cytokine Determinations

To investigate the Th2 profile immune response during the acute phase of the disease, plasma from *S. mansoni*-infected and control animals were processed for cytokine (IL-4, IL-10, IL-13) analyzes by ELISA assay (DuoSet® ELISA Development System, R&D Systems, Minneapolis, MN, USA) according to the manufacturer's instructions. After blood centrifugation (3000 rpm for 10 min at 4°C), plasma was collected and frozen at −80°C for subsequent processing.

For ELISA assay, the plates were incubated with 50 μL of capture antibody, diluted in PBS (0.15 M NaCl) at RT, overnight. Next day, the plates were washed three times with washing buffer (0.05% Tween® 20 in PBS), blocked with 150 μL of PBS-BSA (bovine serum albumin) 10% for 1 h and washed as before. Samples or cytokine patterns (50 μL) were diluted in PBS-BSA and incubated at 4°C, overnight. Next day, the plates were washed as above and incubated with the detection antibody (50 μL) for 2 h at RT, followed by 50 μL of streptavidin conjugated to peroxidase for 20 min protected from exposure to light. After washing, the reaction was revealed by adding 50 μL of substrate [H_2_O_2_: tetramethylbenzidine (TMB)] (1:1) for 20–40 min and blocked with 50 μL of sulfuric acid (H_2_SO_4_ 2N). Samples were read in a SPECTRAMAX 190 Microplate Reader (Molecular Devices, San Jose, CA, USA) at 450 nm.

### Preparation and Quantification of the Peritoneal Lavage Fluid Cellularity

The numbers and types of cells recruited from the bone marrow to the peritoneal cavity were determined by PL. PL fluid was recovered following injection of 5 mL of 0.02M PBS, pH 7.4, and cells were counted in a Neubauer chamber after dilution (1:40) in Turk solution (2% acetic acid solution). Additionally, cytocentrifuged preparations (100 μL of PL fluid/slide) were obtained in a Cytospin 4 Shandon (Thermo Scientific Corporation, Waltham, MA) at 450 rpm for 5 min at room temperature and stained with a routine hematology stain (Panótico Rápido kit, Laborclin, Pinhais, PR, Brazil). Cells (100/slide) were counted in an Olympus BX-41 microscope (Tokyo, Japan) at a magnification of 1,000x.

### Histopathology and Histoquantitative Analyses

Liver samples from uninfected and infected mice (6 animals/group) were removed from the right lobe and divided into approximately 5 mm^3^ fragments, which were immediately fixed in 4% paraformaldehyde in buffered phosphate, pH 7.3, 0.1 M overnight at 4°C (28). Next day, the specimens were transferred to a 0.1 M phosphate buffer solution, pH 7.3 and kept in this solution at 4°C for further histological processing. Samples were then dehydrated, embedded in glycolmethacrylate resin (GMA) (Leica Historesin Embedding Kit, Leica Biosystems, Heidelberg, Germany) as before (28) and cut at 3 μm-thick sections using a Leica microtome RM2155. This histological approach combines optimal fixation and processing for visualization and quantification of inflammatory processes. Three sections per animal were obtained at an interval of 300 μm between sections to ensure analysis of different granulomas. Sections were stained with hematoxylin-eosin (Sigma-Aldrich) for qualitative and quantitative evaluation of granulomas and other parameters.

Histological slides from livers were scanned using a *3D Scan Pannoramic Histech* scanner (3D Histech Kft. Budapest, Hungary) connected to a computer (Fujitsu Technology Solutions GmbH, Munich, Germany). This scanner enables a resolution of 0.23 μm per pixel. Tissue section areas were analyzed using *Pannoramic Viewer 1.15.2 SP2 RTM* (3D Histech kft.) and Histoquant (3D Histech kft.) softwares, which provide a morphometric detailed analysis with precise measurements of different histological parameters, including areas and types of granulomas at high resolution of entire histological slides (19).

The following morphometric parameters were evaluated and quantitated in the liver: (i) Distribution and types of granulomas as previously described (19); (ii) Area taken by granulomas: the total area related to the granulomatous response was measured in three sections per animal. The area occupied by different types of granulomas was also specified. A total of 203 granulomas was recorded. iii) Area taken by non-granulomatous inflammatory infiltrates: the total area occupied by infiltrates was measured in three sections per animal; (iv) Number of parasite eggs in the liver per mm^2^ of tissue; (v) number of eosinophils per total area of granuloma.

Additionally, the proportion of eosinophils was estimated in hepatic granuloma types. For this, the number of eosinophils was counted among 400 immune cells in three randomly chosen granulomas per cell section from each animal. Considering that three sections were studied per animal (*n* = 6 animals), a total of 54 granulomas was analyzed for this eosinophil quantification.

### Conventional TEM

For conventional TEM, hepatic and intestinal (small and large) fragments were prepared as in previous works (19, 29). Tissue was fixed in a mixture of freshly prepared aldehydes [1% paraformaldehyde and 1.25% glutaraldehyde (EM grade, 50% aqueous, Electron Microscopy Sciences-EMS, Hatfield, PA)] in 0.1 M sodium phosphate buffer, pH 7.4, at room temperature (RT). After 2 h, fragments were sliced into small pieces of 1 mm^3^ and fixed in the same fixative overnight at 4°C. Next, the fragments were washed twice in 0.1 M sodium phosphate buffer for 4 h each at 4°C and kept in the same buffer at 4°C for further processing. All fragments were post-fixed in 1% osmium tetroxide in Sym-Collidine buffer, pH 7.4, for 2 h at RT. After washing with sodium maleate buffer, pH 5.2, they were stained en bloc in 2% uranyl acetate (EMS) in 0.05 M sodium maleate buffer, pH 6.0, for 2 h at RT and washed in the same buffer as before prior to dehydration in graded ethanols and acetone, and infiltration and embedding with a propylene oxide-Epon sequence (Eponate 12 Resin; Ted Pella, Redding, CA, USA). After polymerization at 60°C for 16 h, thin sections were cut using a diamond knife on an ultramicrotome (Leica, Bannockburn, IL). Sections were mounted on uncoated 200-mesh copper grids (Ted Pella) before staining with lead citrate and viewed with a transmission electron microscope (CM 10; Philips, or Tecnai G2-20-ThermoFischer Scientific/FEI 2006, Eindhoven, the Netherlands) at 60–80 KV. Electron micrographs from different experiments (*n* = 3) were randomly taken at different magnifications to study the ultrastructural features of secretory granules and other subcellular morphological aspects.

### Tissue Preparation for Immunonanogold Electron Microscopy (immunoEM)

For immunoEM, liver fragments were immediately fixed in fresh 4% paraformaldehyde in phosphate-buffered saline (PBS) (0.02 M sodium phosphate buffer, 0.15 M sodium chloride, pH 7.4) (30). Samples were fixed for 4 h at RT, washed in PBS, immersed in 30% sucrose in PBS overnight at 4°C, embedded in OCT compound (Miles, Elkhart, IN), and stored in −180°C liquid nitrogen for subsequent use.

### Pre-embedding Immunonanogold EM

As detailed before (30), pre-embedding immunolabeling was carried out before standard EM processing (postfixation, dehydration, infiltration, resin embedding and resin sectioning). All labeling steps were carried out at RT on cryosections as before (30) as follows: a) one wash in 0.02 M PBS, pH 7.4, 5 min; (b) immersion in 50 mM glycine in 0.02 M PBS, pH 7.4, 10 min; (c) incubation in a mixture of PBS and BSA (PBS-BSA buffer; 0.02 M PBS plus 1% BSA) containing 0.1% gelatin (20 min) followed by PBS-BSA plus 10% normal goat serum (NGS) (30 min) – (this step is crucial to block non-specific Ab binding sites); (d) incubation with primary Ab (1 h); (e) blocking with PBS-BSA plus NGS (30 min); (f) incubation with secondary Ab (1 h); (g) washing in PBS-BSA (three times of 5 min each); (h) postfixation in 1% glutaraldehyde (10 min); (i) five washings in distilled water; (j) incubation with HQ silver enhancement solution in a dark room according to the manufacturer's instructions (Nanoprobes) (10 min). This step enables a nucleation of silver ions around gold particles. These ions precipitate as silver metal and the particles grow in size facilitating observation under TEM; (k) three washings in distilled water; (l) immersion in freshly prepared 5% sodium thiosulfate (5 min); (m) postfixation with 1% osmium tetroxide in distilled water (10 min); (n) staining with 2% uranyl acetate in distilled water (5 min); (o) embedding in Eponate (Eponate 12 Resin; Ted Pella); (p) after polymerization at 60°C for 16 h, embedding was performed by inverting eponate-filled plastic capsules over the slide-attached tissue sections; and (q) separation of eponate blocks from glass slides by brief immersion in liquid nitrogen. Thin sections were cut using a diamond knife on an ultramicrotome (Leica). Sections were mounted on uncoated 200-mesh copper grids (Ted Pella) before staining with lead citrate and viewed with a transmission electron microscope (CM 10; Philips) at 60 kV. Two controls were performed: (1) primary Ab was replaced by an irrelevant Ab, and (2) primary Ab was omitted. Electron micrographs were randomly taken at different magnifications to study the entire cell profile and subcellular features.

### TEM Quantitative Analysis of Eosinophils

For quantitative studies of secretory granules, electron micrographs randomly taken from eosinophils infiltrated into infected livers were evaluated after conventional preparation for TEM. A total of 148 electron micrographs showing the entire cell profile and nucleus were analyzed to determine the total number of secretory granules per cell section. Moreover, different magnifications from the same cell were taken to identify ultrastructural changes indicative of PMD, classical exocytosis and/or cytolysis. A total of 2,868 secretory granules were counted, and the numbers of intact, emptying and fused granules were established per cell section. Intact granules were observed as membrane-bound organelles full of contents without evident ultrastructural changes. Emptying granules, that is, undergoing losses of their contents, were indicative of the PMD process, and were identified as enlarged granules with lucent areas in their cores, matrices or both and/or disassembled contents in the absence of granule fusions (20, 31). Granules fused with each other and/or with the plasma membrane were indicative of classical exocytosis (32). The process of cytolysis was recognized by partial or total loss of the plasma membrane integrity and/or extracellular deposition of intact granules (31). For controls, tissue eosinophils randomly distributed in the small and large intestine from uninfected animals were also analyzed by TEM.

Additionally, the total number of specific granules positive for MBP-1 was quantitated in electron micrographs obtained from the liver after ultrastructural immunolabeling for this protein. These analyses were done in clear cross-cell sections (total of 9 cells, *n* = 218 granules) exhibiting the entire eosinophil cell profile, intact plasma membranes and nuclei as previously performed for single-cell analyses at high resolution of immunogold-labeled eosinophils (32). All quantitative studies were done using the *Image J* software *(*National Institutes of Health, Bethesda, MD).

Finally, the total number of round large, cytoplasmic vesicles was evaluated per cell section. A total of 755 vesicles was enumerated in 19 randomly taken electron micrographs showing the entire cell profile and nucleus from both infected and uninfected tissue eosinophils.

### Statistical Analyses

ANOVA followed by Turkey multiple comparisons test, or Kruskal-Wallis test was performed using GraphPad Prism version 7.00 for Windows (GraphPad Software, La Jolla California, www.graphpad.com). Significance was *P* < 0.05.

## Results

### The Liver Pathology Induced by *S. mansoni*

Schistosomiasis mansoni is characterized by a robust egg-induced granulomatous inflammation in the liver [reviewed in (33)]. Granulomas are formed around the eggs that are lodged in the presinusoidal capillary venules of this organ; and hepatomegaly, secondary to granulomatous inflammation, occurs early in the evolution of this disease (33). In the mouse model used in the present work, well-characterized granulomas associated with hepatomegaly were clearly observed (Supplementary Figure 1). While the body weights of the infected animals did not show significant difference when compared to the controls (42.7 ± 1.3 g and 40 ± 0.8 g for the infected and control groups respectively, mean ± SEM, *n* = 6 animals/group), the livers were significantly increased (4.3 ± 0.2 g vs. 1.5 ± 0.05 g for infected and control groups respectively, mean ± SEM, *n* = 6 animals/group, *P* < 0.0001).

**Figure 1:**
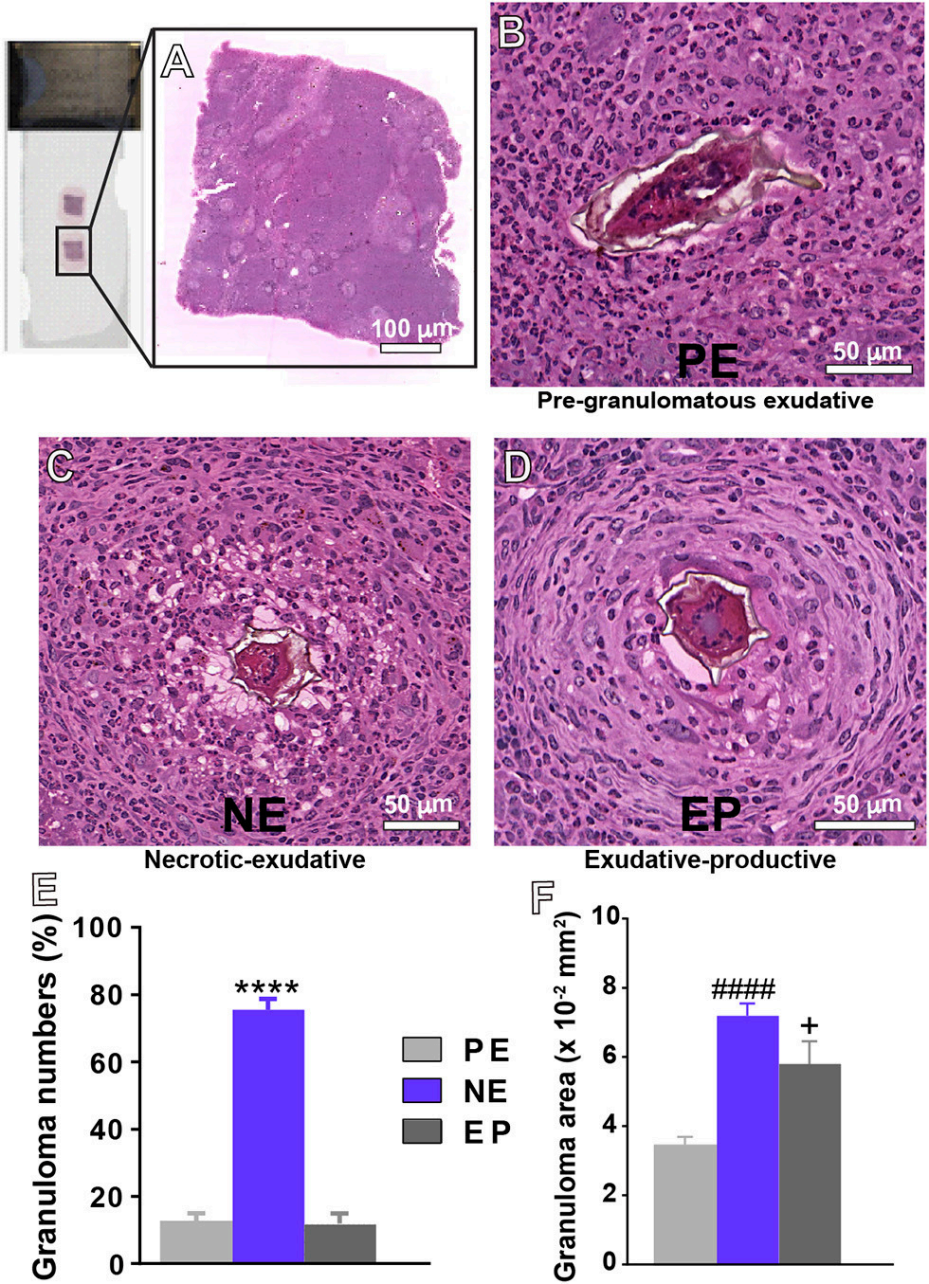
Representative types of granulomas and their frequencies in the livers of mice infected with *S. mansoni*. **(A)** Histological analyses on entire tissue sections identified three types of granulomas: **(B)** Pre-granulomatous exudative (PE), characterized by an infiltrate of inflammatory cells in process of organization around the parasite egg; **(C)** necrotic-exudative (NE), identified by a central halo of necrosis and numerous inflammatory cells distributed irregularly on subsequent layers; and **(D)** exudative-productive (EP), characterized by a rich structure of collagen fibers and inflammatory cells concentrated in the periphery and by a more organized and circumferential aspect. The numbers of granulomas and their areas in the hepatic tissue are shown in **(E,F)**, respectively. Morphometric analyses were performed using Pannoramic Viewer software after whole slide scanning. Data represent mean ± SEM. ****P < 0.0001 vs. numbers of granulomas PE and EP; ^####^*P* < 0.0001 area of granuloma PE; ^+^*P* = 0.04 vs. area of granuloma PE. Images are representative of 3 independent experiments.

To study liver histopathology in more detail, we used a histological approach that combines optimal fixation and processing with a plastic resin (glycolmethacrylate) embedding, which provides better tissue resolution than conventional paraffin embedding (28) with optimal visualization of inflammatory processes including granulomas (27). Sections obtained from glycolmethacrylate-embedded liver fragments were then analyzed by whole slide imaging (WSI), which enables scanning and imaging of entire histological slides (Figure 1A). The resulting digital images have high resolution and offer access to all areas on the slide, thus allowing reliable assessment of the number, evolutional types, frequency and areas of granulomas (19).

By applying WSI, we found three main types of granulomas: pre-granulomatous exudative (PE); necrotic-exudative (NE) and exudative-productive (EP) (Figures 1B–D), as previously demonstrated by our group for this experimental model (19). In the PE stage (Figure 1B), inflammatory cells are in process of organization around the egg while the other stages (Figures 1C,D) are associated with a more organized circumferential structure in which clusters of inflammatory cells such as eosinophils, lymphocytes and macrophages are intermixed with collagen fibers (34, 35). After recording 203 granulomas, we detected that the NE type, identified by a central halo of necrosis and numerous inflammatory cells distributed irregularly on subsequent layers, was the most frequent type of granuloma (Figures 1C–E). Because the sizes of granulomas greatly vary in target tissues, we next measured the tissue area taken by granulomas in the liver. The NE type showed the largest area (Figure 1F). The area occupied by the EP type was significantly higher than that occupied by the PE type (Figure 1F), although these two types of granulomas did not differ in terms of numbers (Figure 1E). Next, the percentage of granuloma area in relation to the entire tissue was obtained. We found that the percentage of hepatic tissue taken by inflammatory granulomatous processes represented 7.7 ± 1.3% (mean ± SEM, *n* = 6 animals) of the liver while the number of parasite eggs in this organ was 2.08 ± 0.5/mm^2^ of tissue. Additionally, we measured the area taken by inflammatory infiltrates (non-granulomatous), which represented 35.1 ± 1.1% (mean ± SEM, *n* = 6 animals). In conjunction, the inflammatory response (non-granulomatous and granulomatous) occupied 42.8 ± 2.4% (mean ± SEM) of the hepatic tissue, thus denoting a remarkably compromised liver.

Accordingly, significant increases in the levels of the serum enzymes alanine aminotransferase (ALT) and aspartate aminotransferase (AST) reflecting altered liver function (7, 36, 37) were detected in response to the infection (Table 1). We observed approximately 3- and 4-fold elevations, respectively, in response to the acute *S. mansoni* infection (Table 1).

**Table 1:** Serum transaminases of uninfected and *S. mansoni*-infected animals.

**Parameters**	**Animals**				*P-*value^**^
	**Uninfected (*n* = 12)**	**Fold ↑**	**Infected (*n* = 12)**	**Fold ↑**	
AST (U/L)	76 ± 12	1.0	322 ± 48	4.24	<0.0001
ALT (U/L)	49 ± 8	1.0	160 ± 24	3.26	<0.0001
De ritis ratio^*^	1.6 ± 0.1	1.0	2.0 ± 0.1	1.25	<0.0001

*AST, Aspartate Aminotransferase; ALT, Alanine Aminotransferase*.

* *De ritis ratio was calculated as [AST/ALT] and indicates the degree of hepatocelular damage*.

** *P-value indicates difference between infected and unifected animals*.

### Detection of Th2 Cytokines in Response to *S. mansoni* Infection

The continuous antigenic stimulation resulting from the trapped eggs in target organs leads to a pronounced inflammatory response at 6–8 weeks post-infection associated with a dominant CD4+ T cell-dependent immune response [reviewed in (38)]. The interleukins (IL) IL-4, IL-10, and IL-13 are dominant cytokines driving this reaction (38). In fact, by analyzing these cytokines in the plasma, we detected significantly higher levels of them in infected compared to uninfected animals (Supplementary Figure 2).

### Eosinophils Are Actively Recruited and Represent the Main Inflammatory Cell Population Within *S. mansoni* Infection-Induced Granulomas

**Figure 2:**
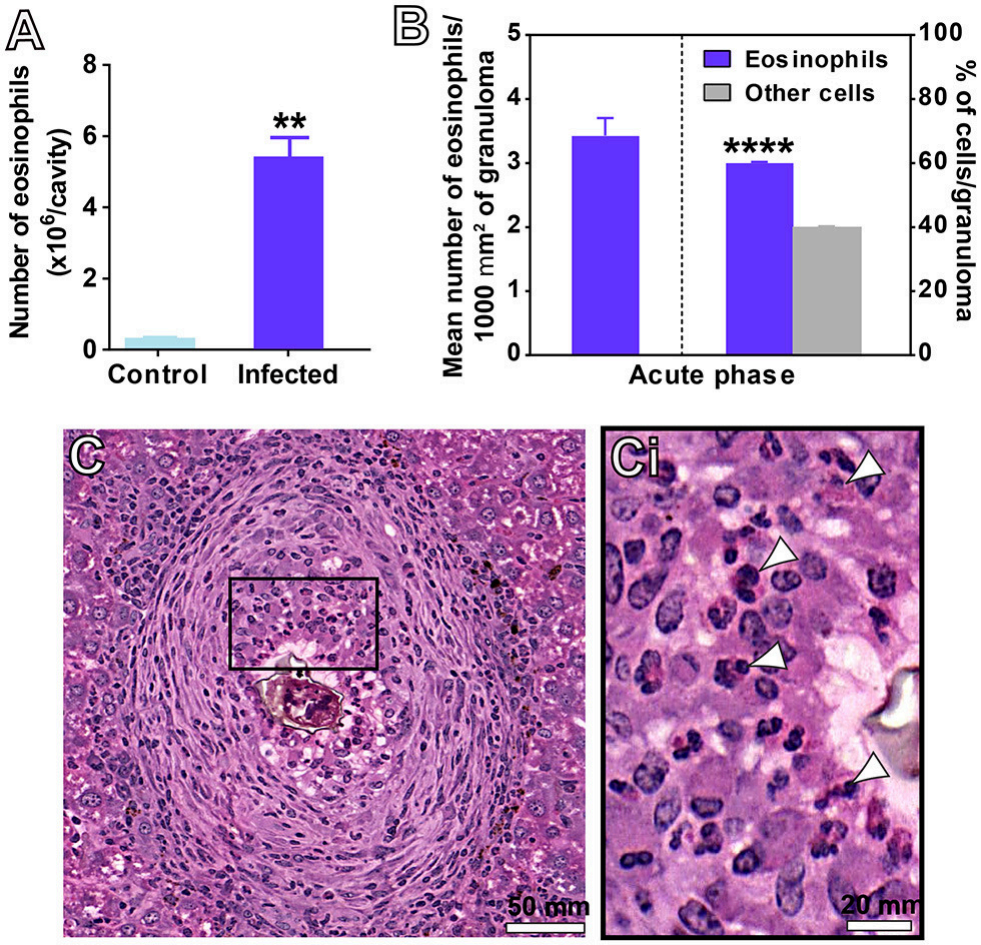
Detection of eosinophils in the peritoneal cavity **(A)** and hepatic granulomas **(B–C)** of mice infected with *S. mansoni*. **(A)** Eosinophil numbers quantitated in the peritoneal lavage. **(B)** Eosinophil numbers quantitated per area of granuloma considering all granulomas, and in the most frequent type of hepatic granuloma (NE type). In **(C)**, a representative NE granuloma. The boxed area in **(C)** is shown in **(Ci)**. Arrowheads indicate examples of eosinophils with characteristic acidophilic cytoplasm. Data represent mean ± SEM. **P = 0.003 vs. control uninfected mice; ****P < 0.0001 vs. other immune cells within granulomas of infected mice. Morphometric evaluations were done with the use of *Histoquant* software. Cytocentrifuged preparations were stained and analyzed at magnification of 1000x. A total of 34,432 eosinophils were counted in 203 granulomas at a magnification of 20x.

During the acute schistosomiasis, increases in the numbers of eosinophils can be detected in the circulation, peritoneal cavity and target tissues (4, 5, 7, 10). Our quantitative analyses of eosinophils in the PL showed that the number of these cells was 15 times higher in the peritoneal fluid collected from infected animals than in controls (4.8 ± 1.1 for infected vs. 0.3 ± 0.02 for control, mean ± SEM, *P* = 0.003, *n* = 4 animals/group) (Figure 2A). Eosinophils were also quantitated within NE granulomas, which, as noted here (Figure 1E) and before (19), represent the most frequent stage of granuloma found in this model of acute schistosomiasis. A total of 34,432 eosinophils was counted. By applying two morphometric evaluations in whole sections, that is, assessment of the eosinophil numbers per granuloma area and proportion of eosinophils per granuloma, we found that these cells corresponded to 60.1 ± 0.3% (mean ± SEM) of all immune cells within NE granulomas (Figures 2B,C,Ci).

### Infiltrating Eosinophils Degranulate Through Different Degranulation Patterns

**Figure 3:**
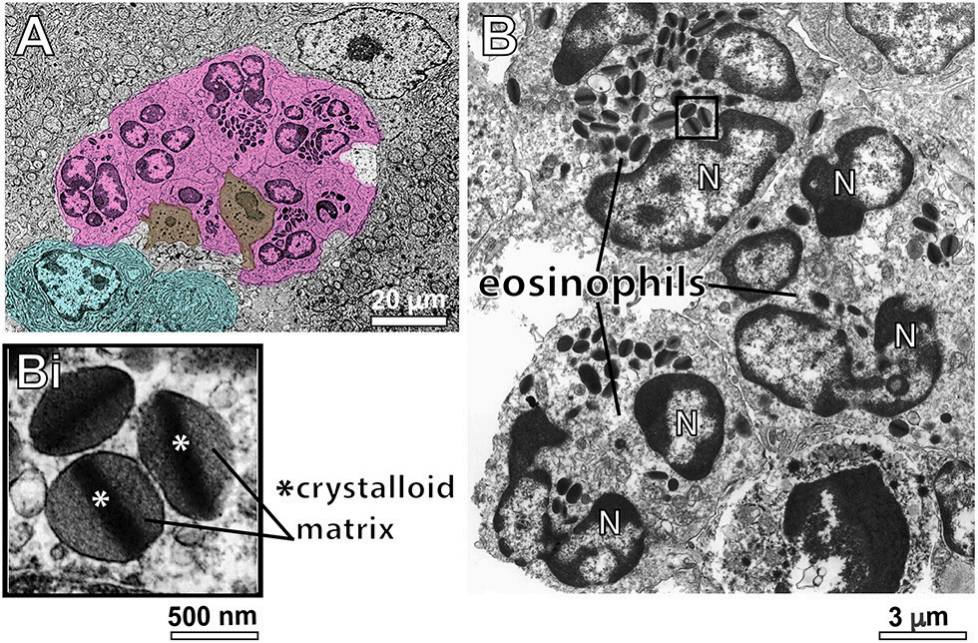
Conventional TEM shows infiltrating eosinophils in the liver of *S. mansoni*-infected mice. **(A)** A representative electron micrograph of the hepatic tissue in low magnification shows a group of eosinophils (colored in pink) in close contact with each other and with neutrophils (brown) and plasma cells (blue). In **(B)**, eosinophils exhibit their typical ultrastructure with a lobulated nucleus (N) and a robust cytoplasmic population of specific granules with a unique morphology-an internal well-defined electron-dense crystalline core and an outer electron-lucent matrix (seen in higher magnification in **Bi**).

Having confirmed the striking number of eosinophils in hepatic granulomas with WSI, we investigated morphological features of these cells at high resolution. For this, liver fragments were processed for conventional TEM. First, eosinophils with typical ultrastructure, that is, exhibiting a polylobed nucleus and a prominent population of cytoplasmic secretory granules with a electron dense crystalloid core surrounded by an electron lucent matrix were easily identified as isolated cells or forming tight groups of cells (Figure 3). Eosinophils were in contact with each other and/or with cells such as neutrophils, plasma cells and lymphocytes (Figure 3).

**Figure 4:**
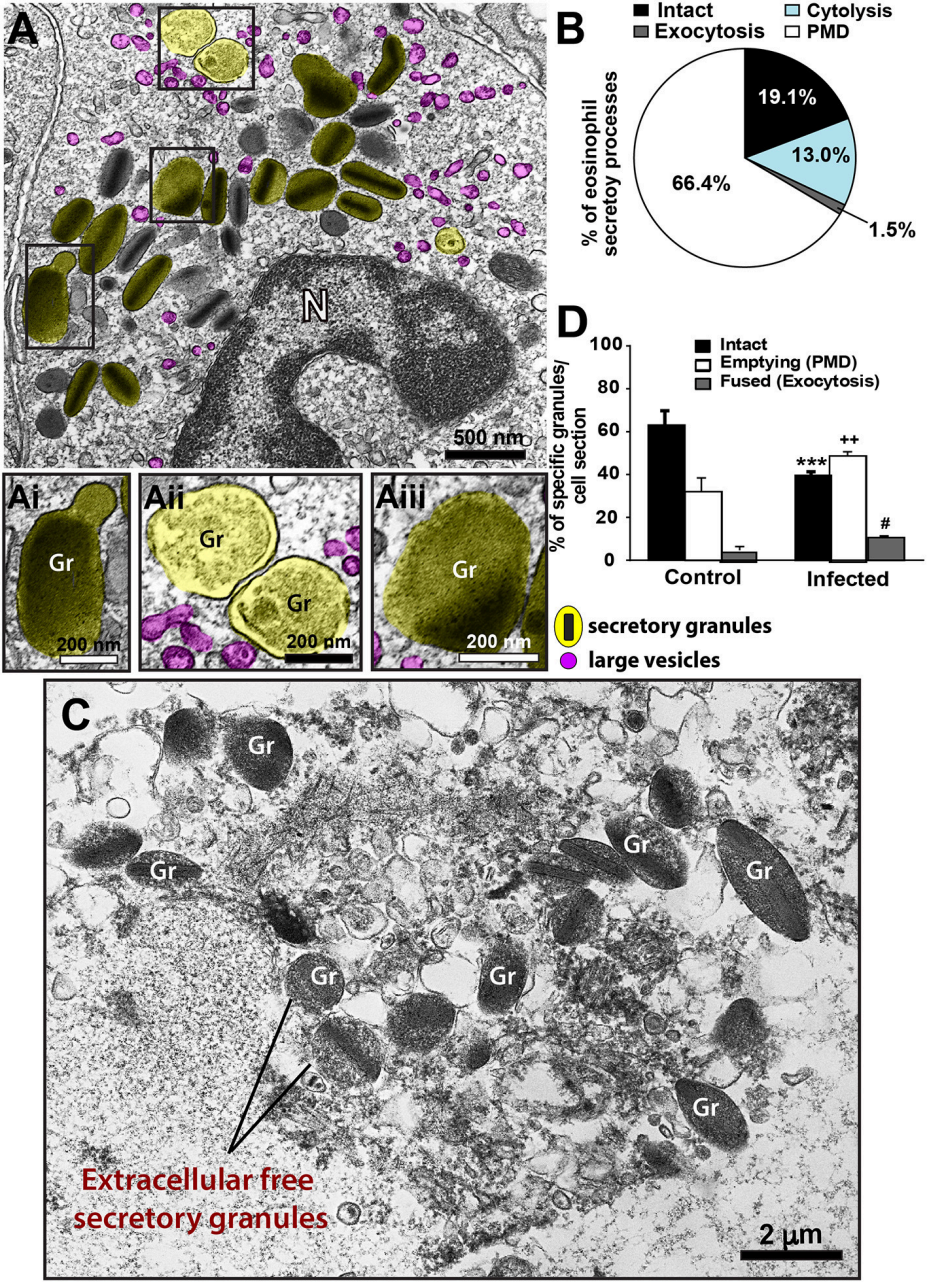
Ultrastructural features of eosinophil degranulation in inflammatory sites of the liver of *S. mansoni*-infected mice. **(A)** A representative eosinophil shows PMD, characterized by the presence of emptying, non-fused secretory granules. The population of eosinophil specific granules is colored in yellow while large vesicles are highlighted in pink. The boxed areas in **(A)** are shown in **(Ai–Aiii)** in higher magnification. **(Ai–Aiii)** Note structural signs of PMD such as granule enlargement and disarrangement of granule cores and matrices. **(B)** Quantification of the secretory patterns shown *in vivo* by hepatic eosinophils in response to the acute infection. In **(C)**, an eosinophil in advanced stage of cytolysis shows extracellular free secretory granules (Gr). **(D)** Most eosinophil secretory granules undergo structural changes indicative of PMD compared to that in uninfected mice. Data represent mean ± SEM. One hundred eight electron micrographs showing the entire cell profile and nucleus were analyzed and 2868 secretory granules were counted. ***P = 0.001 vs. intact granules, ^++^*P* = 0.003 vs. emptying granules, ^#^*P* = 0.03 vs. fused granules of uninfected mice. Gr, secretory granule. *N*, nucleus. Fragments of the liver of animals experimentally infected (acute phase) and intestines (for uninfected controls) were prepared for conventional TEM.

**Figure 5:**
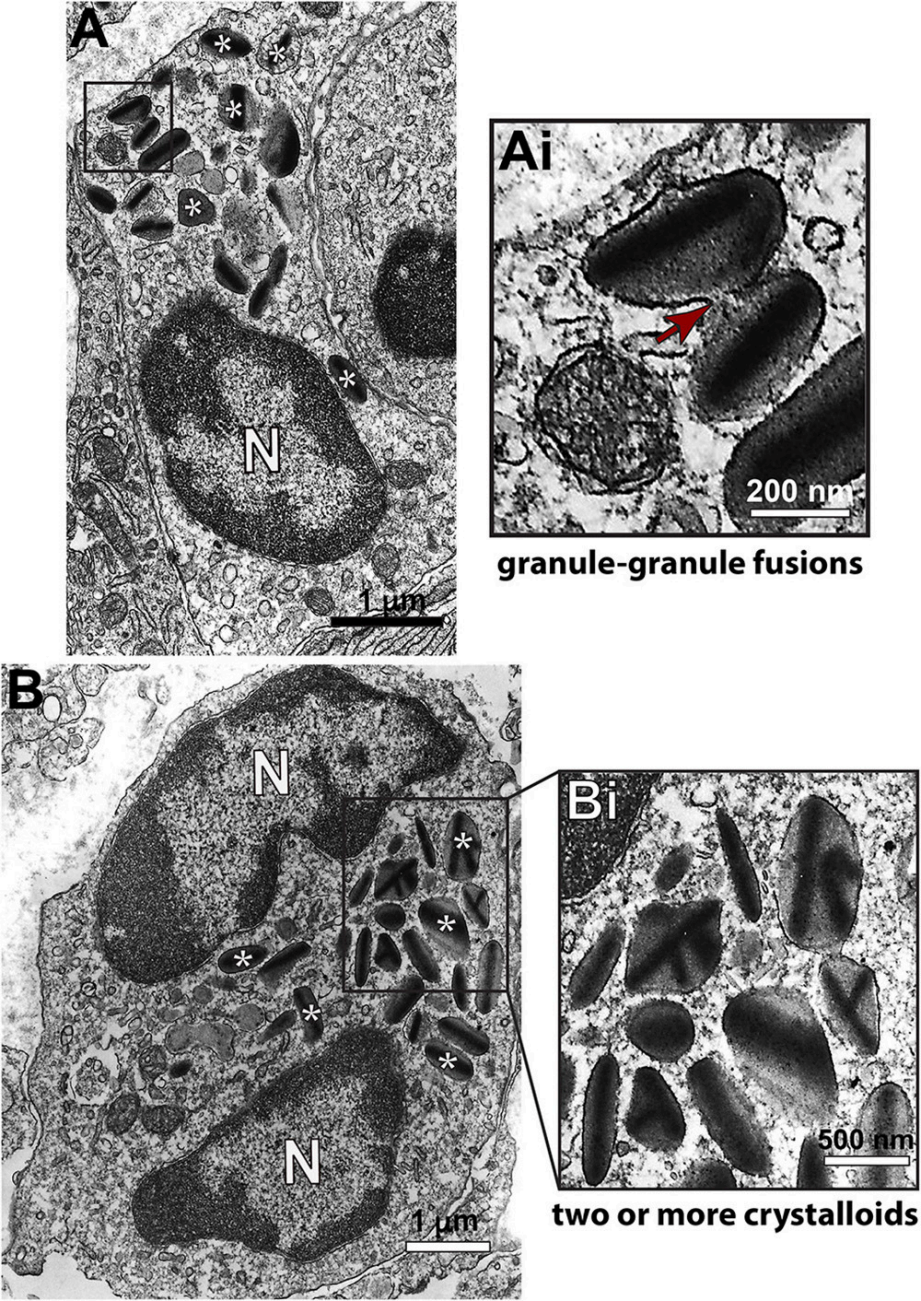
Ultrastructure of eosinophils undergoing compound exocytosis in inflammatory sites of the liver of *S. mansoni*-infected mice. **(A)** A representative electron micrograph shows an eosinophil with granules in process of fusion. In **(B)**, secretory granules show several crystalline cores, a feature also considered as an evidence of fusion. The boxed areas in **(A,B)** are shown in **(Ai,Bi)** in higher magnification. (*) Denotes some of the secretory granules. Arrows indicates a fusion area. *N*, nucleus. Fragments of the liver of animals experimentally infected (acute phase) were prepared for conventional TEM.

Second, we sought to identify and characterize ultrastructural signs underlying degranulation (as described in material and methods) in infiltrated eosinophils. We found that while 19.1% of eosinophils were intact (Figure 4A), most eosinophils (66.4%) exhibited predominantly features of PMD (Figures 4Ai–iii,B). Characteristic features of cytolysis (Figure 4C) were observed in 13.0% of eosinophils (Figure 4B) and just 1.5% (Figure 4B) showed classical or compound exocytosis (Figure 5).

Third, we wondered if the numbers of intact and emptying secretory granules in infected eosinophils would differ from values shown by resting cells. Because eosinophils are not normally found in the liver, we analyzed the ultrastructure of eosinophils distributed in the intestinal tract of uninfected animals (Supplementary Figure 3). A total of 131 cells showing intact plasma membranes, that is, not undergoing cytolysis, was evaluated. Our quantitative analysis revealed a significant increase of emptying, non-fused granules (49.5 ± 1.9/cell section for infected vs. 32.3 ± 6.9/cell section for control, mean ± SEM, *P* = 0.003, *n* = 2868 secretory granules) and a significant reduction of intact granules in response to the acute schistosomiasis (*P* = 0.001) (Figure 4D), thus confirming the ability of these cells to secrete mostly through PMD.

### PMD Is the Predominant Secretory Process of MBP-1 Release by *S. mansoni*-Activated Eosinophils

MBP-1 is the main cationic protein stored as preformed pool within eosinophil secretory granules and considered a hallmark for these cells. Because MBP-1 has been associated with the immunopathogenesis of various helminthic diseases, including schistosomiasis mansoni [reviewed in (39)], we next investigated the structural mechanism of MBP-1 release by applying pre-embedding immunonanogold EM. This methodology has proved to be very effective in localizing immune mediators in human eosinophils and other cells from the immune system (30, 31, 40).

**Figure 6:**
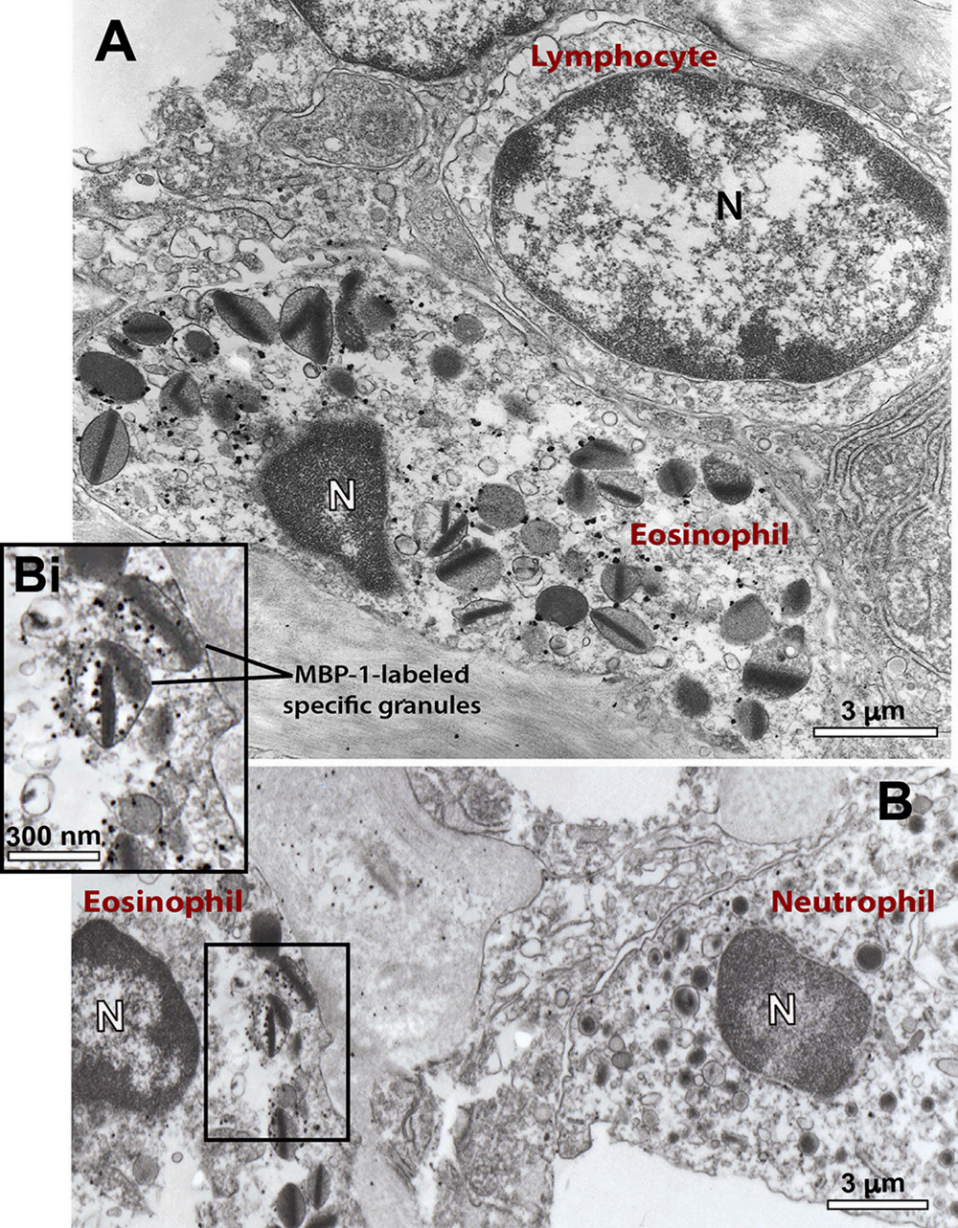
Ultrastructural immunolocalization of MBP-1 in the liver from a *S. mansoni*-infected mouse. **(A,B)** Positivity is seen within representative infiltrating eosinophils while other inflammatory cells such as lymphocyte **(A)** and neutrophil **(B)** are negative. Labeling was associated with secretory (specific) granules, as observed in higher magnification **(Bi)**. A close apposition between an eosinophil and a lymphocyte is noted in **(A)**. Liver fragments were prepared for pre-embedding immunonanogold electron microscopy. *N*, nucleus.

**Figure 7:**
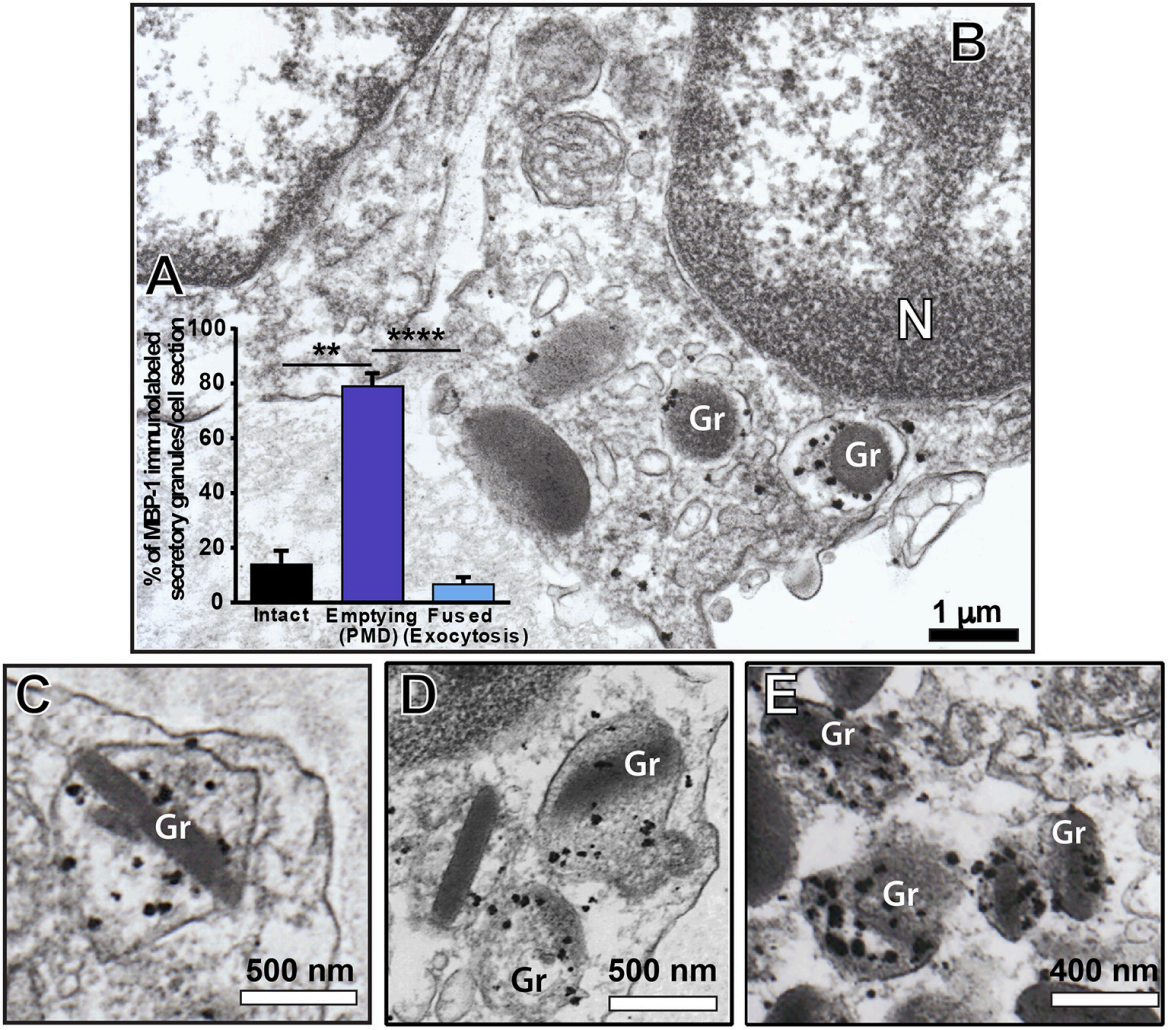
Immunolocalization of MBP-1 on liver-infiltrating eosinophils from *S. mansoni*-infected mice. **(A)** Most MPB-1-positive secretory granules (~80%) showed characteristics of piecemeal degranulation (PMD). **(B–E)** Single-cell analyses at high-resolution reveal robust labeling of MBP-1 within secretory granules (Gr) of activated eosinophils. Note the typical signs of PMD such as enlargement and disarrangement of granule cores and matrices. *N*, nucleus. Data represent mean ± SEM. The numbers of labeled and not labeled granules (*n* = 218 granules) were counted in electron micrographs (*n* = 9). **P = 0.005 vs. intact granules; ****P < 0.0001 vs. fused granules. Liver fragments were prepared for pre-embedding immunonanogold electron microscopy.

We observed a clear labeling for MBP-1 in the entire population of inflammatory eosinophils while other infiltrated immune cells were completely negative (Figure 6). Infected cells, for which the primary antibody was replaced by an irrelevant antibody or omitted (Supplementary Figure 4), were negative. MBP-1 positivity was mostly associated with eosinophil secretory granules (Figure 7). By using software for quantitating granules, we detected that the majority of them (84.0 ± 2.5%, mean ± S.E.M, *n* = 9 cells) in each eosinophil section was labeled for MBP-1 and that most of these labeled granules were undergoing PMD (Figure 7A), that is, showing disassembled cores, enlargement, matrix coarsening and/or reduced electron-density (Figures 7B–E).

### Identification of a Vesicular Trafficking of MBP-1 Within *S. mansoni*-Triggered Eosinophils in the Liver

**Figure 8:**
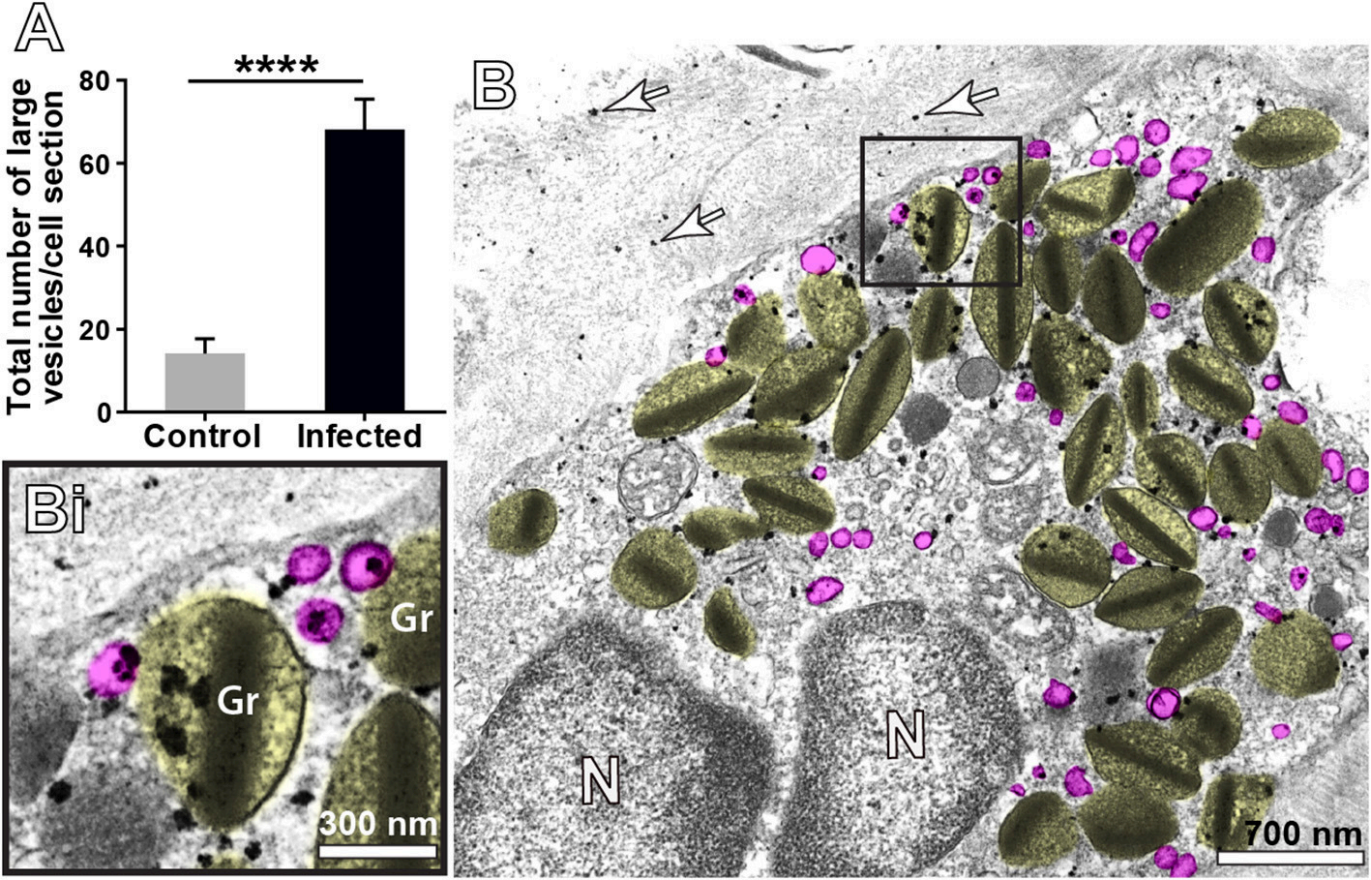
Vesicular trafficking of MBP-1 in the cytoplasm of inflammatory eosinophils in the liver of *S. mansoni*-infected mice. **(A)** The acute infection induces a prominent formation of cytoplasmic, large (80–150 nm) round vesicles (highlighted in pink in **B**. Note in **Bi**) that immunolabeling for MBP-1 is clearly associated with several of these vesicles in addition to secretory granules (Gr, highlighted in yellow). Arrows indicate extracellular deposition of MBP-1. Data represent mean ± SEM. The numbers of vesicles (*n* = 755) were counted in a total of 19 electron micrographs, after conventional processing for TEM. ****P < 0.0001 vs. control group. For immunolabeling of MBP-1, liver fragments were prepared for pre-embedding immunonanogold electron microscopy.

When human eosinophils are activated by inflammatory stimuli, there is a significant increase of cytoplasmic large vesicles termed Eosinophil Sombrero Vesicles (EoSVs), which are involved in the transport of granule-derived products (15, 32, 41). The existence of EoSVs-like vesicles in mouse eosinophils is unknown. By performing conventional TEM in the liver from infected animals, we noticed, for the first time, the presence of numerous round, reasonably large (~80–150 nm) vesicles in the cytoplasm of inflammatory eosinophils (highlighted in pink in Figures 4A, 8B). By enumerating a total of 755 vesicles in infected and uninfected tissue eosinophils, we found a significant increase of these vesicles in infected compared to uninfected cells (68.1 ± 7.3/cell section for infected vs. 14.2 ± 3.5/cell section for control, mean ± SEM, *P* < 0.0001, *n* = 19 cells) (Figure 8A).

Next, we analyzed the population of these vesicles after ultrastructural immunolabeling for MBP-1. Our single-cell analyses at high resolution were revealing in demonstrating MBP-1-positive vesicles distributed in the cytoplasm and attached to or surrounding the surface of emptying granules (Figure 8B). Computational analyses showed that ~20% of all cytoplasmic vesicles from the same size range were carrying MBP-1 (Figure 8Bi). Altogether, our findings consistently demonstrate the occurrence of a secretory process based on vesicular trafficking as a main mechanism for MBP-1 release in response to the acute schistosomiasis in mice. Accordingly, MBP-1 immunolabeling was also detected in the extracellular matrix (Figure 8B, arrows). Interestingly, the deposits of MBP-1 were not massive, but dispersed in the extracellular matrix (Figure 8B, arrows).

## Discussion

Expansion and recruitment of eosinophils is a central feature of the host response to the *S. mansoni* infection. How these cells release their products in target organs of this disease is unknown. Our study describes marked eosinophil degranulation *in vivo* in the liver triggered by schistosomiasis mansoni in mice and identifies, for the first time, PMD as the main mode of eosinophil secretion. We also provide direct evidence that MBP-1 is transported in the cytoplasm of infiltrating eosinophils and released through a vesicular trafficking in response to the acute infection.

In this work, we explore the ability of inflamatory eosinophils to degranulate in a murine model of schistosomiasis mansoni. Acute schistosomiasis is characterized by a systemic hypersensitivity reaction against the migrating schistosomula and eggs (42). During the first 3–5 weeks, the host is exposed to migrating immature parasites while at weeks 5–6, the parasite matures and begins to produce eggs, which is associated with a Th2 response (reviewed in 39). All classical parameters confirmatory of the acute infection, such as hepatomegaly, high density of eggs in a target organ; well-characterized granulomatous inflammation around deposited eggs; alteration of liver enzymes, increased levels of Th2 cytokines and extensive eosinophil infiltration were consistently demonstrated in this model. Moreover, our detailed analysis of granuloma formation in infected mice corroborates the prevalence of the NE granuloma, which is greatly enriched in inflammatory cells and it is also the major granuloma type found during the acute schistosomiasis mansoni in humans (42).

Acute schistosomiasis in mice led to substantial degranulation of infiltrating eosinophils in the liver through PMD. PMD is a frequent secretory process of human eosinophils observed *in vivo* in varied human inflammatory and other disorders such as asthma (43); nasal polyposis (44); allergic rhinitis (44, 45); ulcerative colitis (44); Crohn's disease (44); atopic dermatitis (46); functional dyspepsia (47), gastric carcinoma (48); shigellosis (49) and cholera (50), but this is the first time that it is clearly recognized during the acute infection with *S. mansoni*. In this mode of secretion, eosinophils release granule contents, but retain their granule containers [reviewed in (51)]. PMD is identified mainly by structural disarrangement of the granules cores and matrices within granules delimited by intact membranes, but other subtle signs such as granule matrix coarsening and granule enlargement can additionally indicate PMD occurrence in mouse eosinophils (52) (Figure 4). In fact, there have been controversies that mouse eosinophils are not able to “degranulate” in some mouse models of allergic airway inflammation (24, 53, 54). In our work using detailed ultrastructural analyses to examine mouse eosinophils in a “classic” host-response model of murine schistosomiasis, we provide definitive findings for the *in vivo* capacity of mouse eosinophils, like human eosinophils, to undergo PMD.

To compare the extent of the secretory processes shown by infiltrating eosinophils in the liver of infected animals, we used resident intestinal eosinophils as controls since eosinophils are not resident cells of the liver. Hepatic inflammatory eosinophils had significantly higher numbers of secretory granules with PMD features compared to the eosinophil population of the intestinal tract (Figure 4D). However, it should be highlighted that this population showed considerable PMD (Figure 4D), which can be explained by the fact that intestinal eosinophils are phenotypically distinct from blood eosinophils and exhibit an activated phenotype based on their cytokine expression and degranulation status (55). In fact, eosinophils that reside in the gastrointestinal tract are required for the homeostatic intestinal immune responses, including IgA production through secretion of cytokines (56, 57) and constitutively express antigen presentation markers (58). Thus, intestinal eosinophils are more active and consequently with a higher activity of secretion.

In addition to PMD, our quantitative EM analyses revealed that 13% of liver-infiltrating eosinophils exhibited different degrees of cytolysis (Figures 4B,C), which deposits cell-free secretory granules in the surrounding tissue [reviewed in (31)]. Cytolysis is a physiologically important mode of eosinophil secretion because the specific granules remain active even after cell death (31). Cytolysis is defined ultrastructurally by physical rupture of the cell and is morphologically distinct from both apoptosis and necrosis (46, 59). More recently, another form of cell death–pyroptosis–, which is mediated by caspase-1 (60), was identified in hepatic eosinophils isolated from a mouse model of *S. mansoni* infection (61). Because cell disruption is also a feature of pyroptotic cells (60), we cannot rule out that part of the cytolytic eosinophil population found by our EM analyses may be undergoing pyroptosis.

Formation of large secretory vesicles (EoSVs) that arise from eosinophil specific granules and contain granule proteins and cytokines is another indication of PMD and has been documented in human eosinophils [reviewed in (51, 62)] but heretofore not in mouse eosinophils. Moreover, amplified formation of EoSVs is considered a morphological feature of activated human eosinophils (15, 41). For example, eosinophils stimulated with CCL11/eotaxin-1 or tumor necrosis factor alpha (TNF-α) show increased numbers of cytoplasmic EoSVs (40) as well as do naturally activated eosinophils from patients with hypereosinophilic syndrome when compared to normal donors (63). Our present quantitative EM studies demonstrate, for the first time, that *S. mansoni*-triggered mouse eosinophils have an augmented population of large (80–150 nm) round vesicles, analogously to EoSVs found in human eosinophils (15). As with human EoSVs, these large vesicles from mouse eosinophils are seen distributed in the cytoplasm and clearly associated with secretory granules, but these vesicles do not exhibit the same typical tubular morphology of human EoSVs (Figures 4A,Aii, 8B,Bi).

MBP has been extensively detected extracellularly in inflammatory sites of eosinophil-associated human diseases (64–68), including schistosomiasis mansoni (69). Here, we also observed extracellular localization of MBP in the liver elicited by the acute *S. mansoni* infection in mice (Figure 8B). MBP extracellular deposition comes from degranulating eosinophils, which can release their products via, as noted, cytolysis, classical/compound exocytosis and/or PMD. Because PMD was detected in most infiltrated eosinophils (66.4%) and relies on vesicular transport of granule products, we were expecting to find granule-mobilized MBP in association with cytoplasmic vesicles. Indeed, our single-cell analyses using very small gold particles (1.4 nm) for membrane microdomains access enabled labeling of MBP-1 on intracellular vesicles of eosinophils in the livers of *S. mansoni*-infected animals (Figures 7, 8). Therefore, it is clear that part of the cytoplasmic vesicle population is trafficking MBP-1 within inflammatory eosinophils for extracellular release. In fact, deposits of MBP-1 were dispersed in the extracellular matrix (Figure 8), which is compatible with gradual vesicle-mediated release of this protein. Vesicular secretion of MBP was also described in human activated eosinophils *in vitro* (63), but this is the first report on this secretory pathway in mice and in association with a parasitic disease *in vivo*.

What is the meaning of PMD and the release of MBP-1 through PMD during acute shistosomiasis? Eosinophils produce an array of granule-stored immune mediators that are known to be key regulators in diverse physiological and pathological processes (3) and are a source of both pro-inflammatory and immune regulatory cytokines (70). In contrast to classical and compound exocytosis, whereby whole granule contents are extruded *in toto*, PMD enables extracellular delivery of specific mediators in small amounts (“piece-by-piece”) through a vesicular trafficking. Gradual release of immune mediators, including MBP-1, by inflammatory eosinophils may be involved with immunoregulatory functions of these cells during schistosomiasis. MBP, in addition to be classically associated with parasite killing, has been implicated with the regulation of cytokine responses during helminth infections (71).

In fact, there is increasing evidence that eosinophils exert a role of immunoregulation in both adaptive and innate immunity including in the context of parasitic diseases [reviewed in (12, 72)]. It is now well documented that eosinophils have key immunoregulatory functions as professional antigen-presenting cells and as modulators of CD4(+) T cell, dendritic cell, B cell, mast cell, neutrophil, and basophil functions(72). Of interest, our comprehensive ultrastructural analyses revealed direct contact of eosinophils undergoing PMD with other immune cells such as neutrophils (Figure 3A), plasma cells (Figure 3A), and lymphocytes (Figure 6A). It is documented that eosinophils are able to modulate the functions of other leukocytes [reviewed in (2)] and our findings showing eosinophil interactions with other immune cells may represent such capacity.

Our results shed light on the ill-understood *in vivo* roles of eosinophils, underlined by their degranulation ability, in target organs of the *S. mansoni* infection. The current view of eosinophils as effector cells, able to kill parasites through massive discharge of granule products, is beginning to change. However, the meaning of eosinophil cell-to-cell interactions and if PMD represent a subtle immunomodulatory contribution of eosinophils in both experimental and human schistosomiais awaits further investigation.

Lastly, it is important to highlight the emerging role of eosinophils in tissue homeostasis and repair (3). Sustained PMD-mediated secretion of infiltrating eosinophils during acute schistosomiasis might additionally be associated with the competence of these cells to promote a tissue repair response. Particularly in the liver, the functions of eosinophils and type-2 cytokine signaling were studied in the context of experimental tissue regeneration (73). It was demonstrated that type 2 immune responses related to eosinophils and IL-4/IL-13, via IL-4Rα in hepatocytes, stimulated liver regeneration after experimental injury (73). Thus, it is clear that eosinophils have unanticipated functions *in vivo* and their roles during eosinophilic diseases such as schistosomiasis have been more difficult to establish.

In summary, our findings identify PMD as the main secretory process of inflammatory eosinophils in the liver of S*. mansoni*-infected mice, with detection of granule-derived vesicular transport of MBP-1 in response to the infection. Vesicle-mediated release of MBP-1 and other immune mediators, such as cytokines stored within eosinophil granules may be associated with an immunoregulatory function of eosinophils, but the definitive roles of these cells in the parasitic immune response remains to be recognized. Our present work also expands our understanding of the ultrastructural aspects of mouse eosinophils, their ability to degranulate and the basic mechanisms that underlie the functioning of these cells in this experimental model.

## Author Contributions

RM provided the study supervision and prepared the manuscript. FD, KA, KM, and TS performed experiments. FD prepared the figures. GSCR, FD, VC, and GOLR performed cytokine analyses. RM, PW, HC-G, and FR provided critical editing of the manuscript. All authors contributed in part to writing and editing the manuscript and approved the final version.

### Conflict of Interest Statement

The authors declare that the research was conducted in the absence of any commercial or financial relationships that could be construed as a potential conflict of interest.
