# Supplementary material for: Identification of Piecemeal Degranulation and Vesicular Transport of MBP-1 in Liver-Infiltrating Mouse Eosinophils During Acute Experimental Schistosoma mansoni Infection
Source: Front Immunol. 2018 Dec 20;9:3019. doi: 10.3389/fimmu.2018.03019 (PMC6306457; doi:10.3389/fimmu.2018.03019)
Supplement: Supplementary file 1 [file Image_1.pdf]

Supplementary Material

Identification of piecemeal degranulation and vesicular transport of MBP-1 in liver-infiltrating mouse eosinophils during acute experimental *Schistosoma mansoni* infection

Felipe F. Dias[1], Kátia B. Amaral[1], Kássia K. Malta[1], Thiago P. Silva[1], Gabriel S. C. Rodrigues[1], Florence M. Rosa[2], Gisele O. L. Rodrigues[3], Vivian V. Costa[4,5], Hélio Chiarini-Garcia[6], Peter F. Weller[7] and Rossana C.N. Melo[1, 7*]

[1]Laboratory of Cellular Biology, Department of Biology, Federal University of Juiz de Fora, MG, Brazil.

[2]Laboratory of Parasitology, Department of Parasitology, Microbiology and Immunology, Federal University of Juiz de Fora, MG, Brazil

[3]Laboratory of Immunopharmacology, Department of Biochemistry and Immunology, Federal University of Minas Gerais, MG, Brazil.

[4]Center for Drug Research and Development of Pharmaceuticals, Federal University of Minas Gerais, MG, Brazil.

[5]Research Group in Arboviral Diseases, Department of Morphology, Federal University of Minas Gerais, MG, Brazil.

[6] Laboratory of Reproduction and Structural Biology, Department of Morphology, Federal University of Minas Gerais, MG, Brazil.

[7] Division of Allergy and Inflammation, Department of Medicine, Beth Israel Deaconess Medical Center, Harvard Medical School Boston, Massachusetts, USA.

*Correspondence: Dr. Rossana C. N. Melo
Federal University of Juiz de Fora
Department of Biology, Laboratory of Cellular Biology
Rua José Lourenço Kelmer, Juiz de Fora, MG, 36036-900, Brazil
rossana.melo@ufjf.edu.br

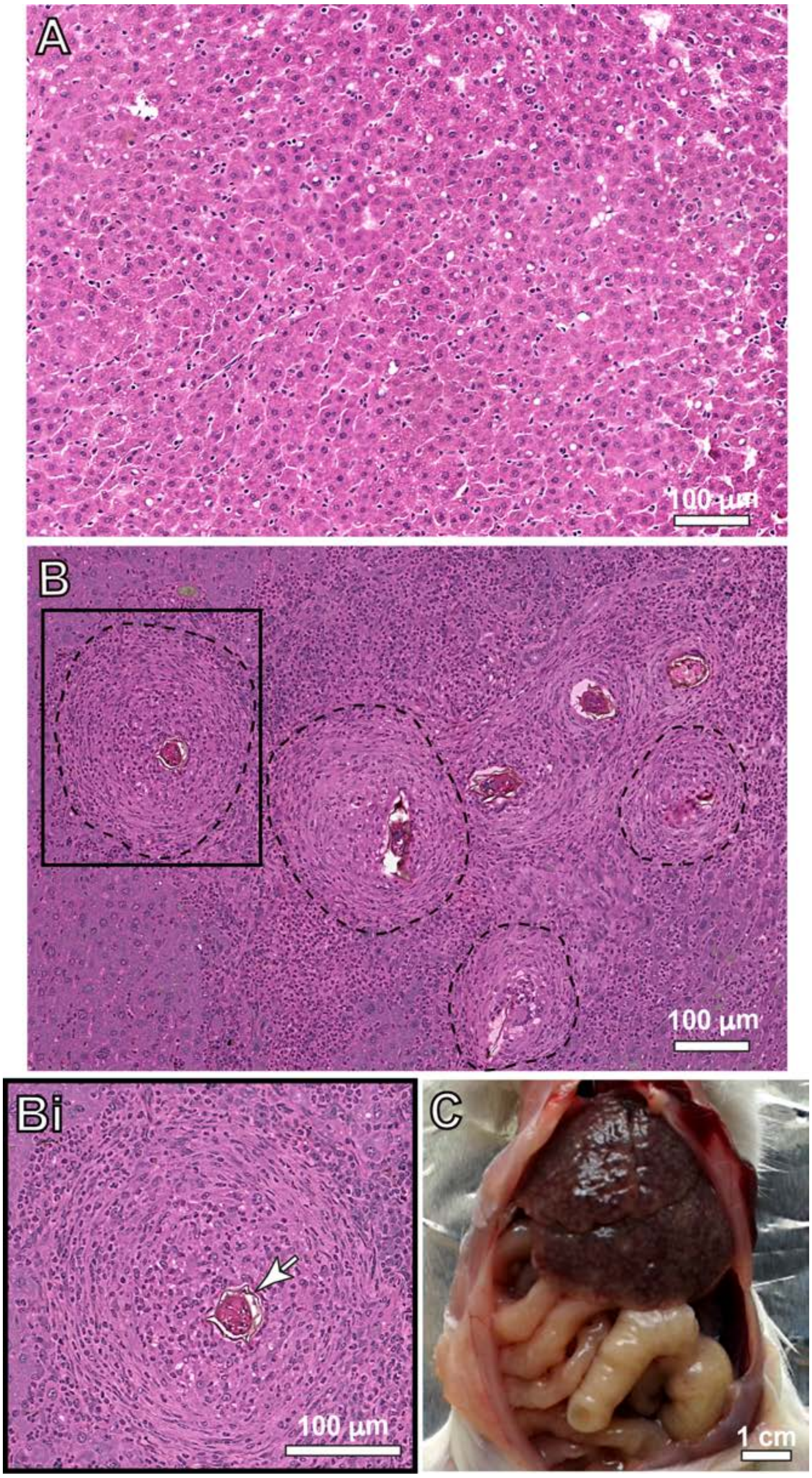

Supplementary Figure 1. Representative histological images of the liver from uninfected (A) and *S. mansoni*-infected mice (B). A normal hepatic tissue is seen in (A) while typical granulomas at different developmental stages (circles) are observed in (B). One mature granuloma characterized by a central parasite egg (arrow) surrounded by a dense population of inflammatory cells is shown in high magnification in (Bi). In (C), typical hepatomegaly in an infected animal. Fragments (n=6 animals from each group) were fixed in buffered paraformaldehyde, embedded in glycolmethacrylate resin and cut into 3 µm-thick sections, which were stained with hematoxylin-eosin.

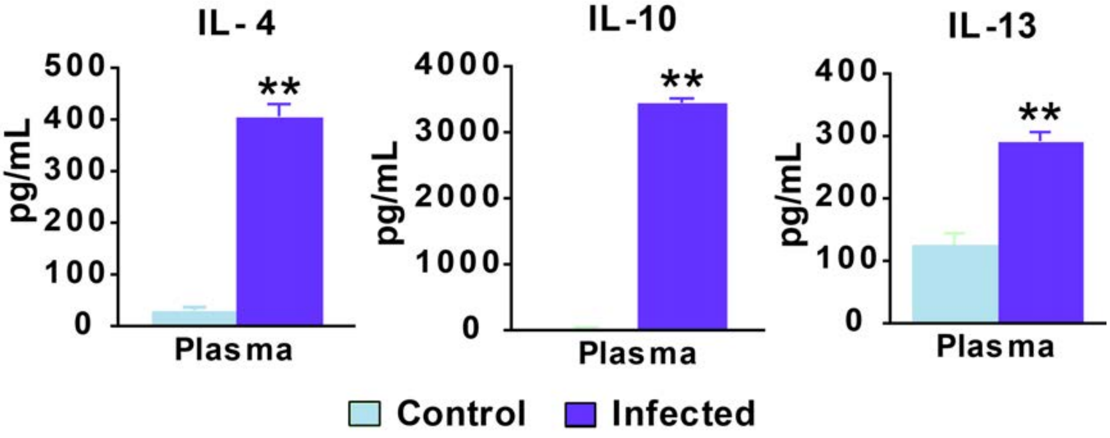

Supplementary Figure 2. Plasma Th2 cytokine determinations. Plasma levels of interleukin-4 (IL-4), interleukin-10 (IL-10) and interleukin-13 (IL-13) in uninfected and *S. mansoni*-infected mice are shown in (A-C), respectively. Data represent mean ± SEM. n= 6 animals/group. ** $P = 0.004$ versus plasma levels of IL-4 and IL-13 of uninfected mice and ** $P = 0.002$ versus plasma levels of IL-10 of uninfected mice.

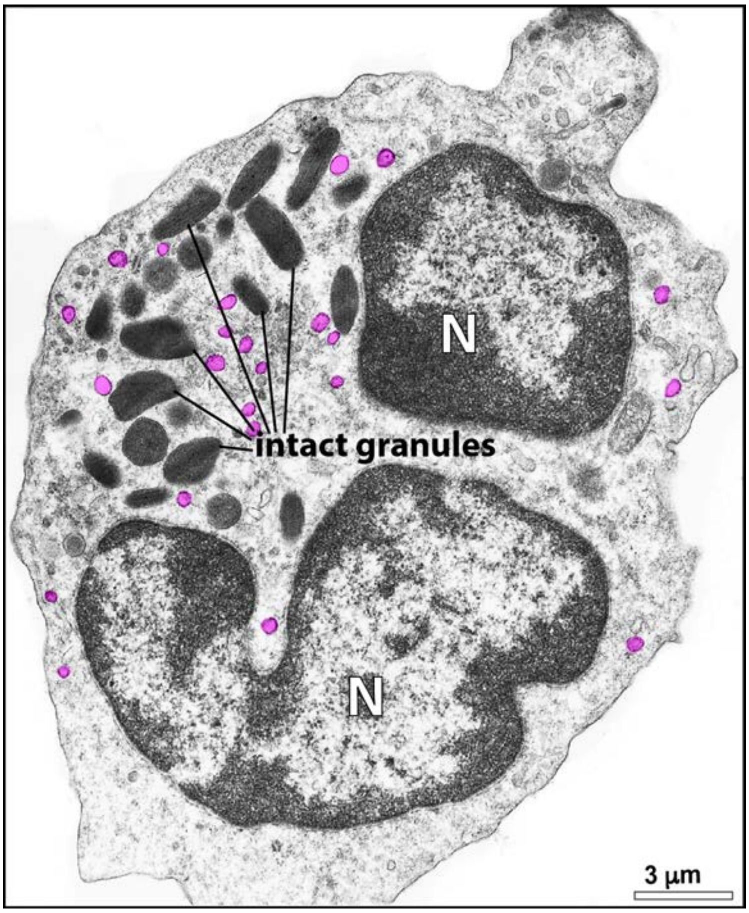

Supplementary Figure 3. A representative electron micrograph from a resting eosinophil in the intestinal tract. The typical eosinophil ultrastructure with intact secretory granules (Gr) and lobulated nucleus (N) is observed. Cytoplasmic large vesicles were highlighted in pink. Fragments of the intestinal tract were processed for conventional TEM.

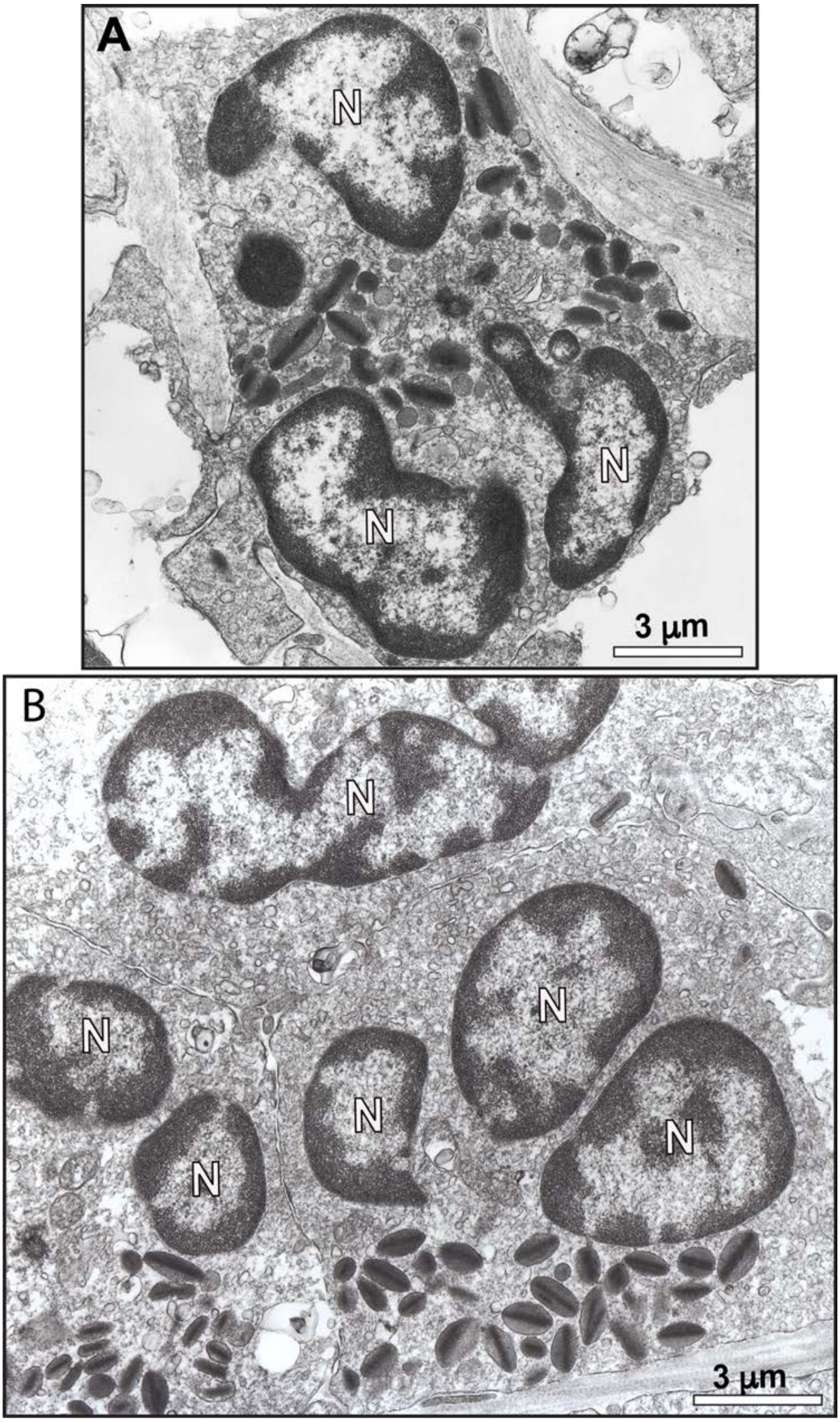

Supplementary Figure 4. Tissue eosinophils from *S. mansoni*-infected mice observed after pre-embedding immunonanogold electron microscopy. In (A), a representative electron micrograph in which the primary antibody was replaced by an irrelevant antibody. In (B), a representative electron micrograph in which the primary antibody was omitted. Note negative immunolabeling in both images. N, nucleus. Fragments of the liver of animals experimentally infected (acute phase) were prepared for pre-embedding immunonanogold electron microscopy and eosinophils infiltrated in this organ were analyzed.
